# All-cause and cause-specific mortality in patients with depression in Scotland

**DOI:** 10.1192/j.eurpsy.2023.996

**Published:** 2023-07-19

**Authors:** R. Alotaibi, N. Halbesma, S. Wild, C. A. Jackson

**Affiliations:** Usher Institute, University of Edinburgh, Edinburgh, United Kingdom

## Abstract

**Introduction:**

Premature mortality in people with depression is well established. A better understanding of the causes of death and the relative risks of death from each cause may help identify factors that contribute to the health inequalities between people with and without depression.

**Objectives:**

To describe all-cause and cause-specific mortality of people with a hospital admission record for depression in Scotland, relative to the general population.

**Methods:**

We used a linked population-based dataset of all psychiatric hospital admissions in Scotland to the national death dataset to identify 28,837 adults ≥18 years of age who had a hospital admission record of depression between 2000 and 2019. We obtained general population estimates and mortality data from the National Records of Scotland and quantified the relative difference in mortality by calculating the standardised mortality ratio (SMR), using indirect standardisation and stratifying by sex.

**Results:**

During a median follow-up of 8.1 years, 7,931(27.5%) people who were hospitalised for depression died. Circulatory system diseases were the most common causes of death. Standardised all-cause mortality was more than three times higher than would be expected based on death rates in the general Scottish population. SMRs were similar in men and women for all-cause mortality and, in general, for cause-specific death (Table 1). The SMR for the suicide category was markedly higher in women than men, partly explained by the higher suicide mortality in males than females in the general population.

**Table 1:** All-cause and cause-specific mortality of people hospitalised for depression in Scotland 2000-19

	**Observed deaths, n** **(All)**	**Expected deaths, n** **(All)**	**SMR (95% CI)** **(All)**	**Observed deaths, n** **(Male)**	**Expected deaths, n** **(Male)**	**SMR (95% CI)** **(Male)**	**Observed deaths, n** **(Female)**	**Expected deaths, n** **(Female**	**SMR (95% CI)** **(Female)**
** All-cause**	7,931	2427	3.3(3.2-3.3)	3617	1052	3.4(3.3-3.5)	4314	1375	3.1(3.0-3.2)
**Circulatory system diseases**	2,020	806	2.5(2.4-2.6)	886	343	2.6(2.4-2.7)	1,134	463	2.4(2.3-2.6)
**Neoplasms**	1,153	682	1.7(1.6-1.8)	534	306	1.7(1.6-1.9)	619	376	1.6(1.5-1.8)
**Respiratory system diseases**	1,106	292	3.8(3.6-4.0)	453	112	4.0(3.7-4.4)	653	180	3.6(3.3-3.9)
**Mental & behavioural disorders**	835	131	6.4(5.9-6.8)	333	52	6.4(5.7-7.2)	502	79	6.3(5.8-6.9)
**Accidents**	395	69	5.7(5.2-6.3)	224	38	5.9(5.1-6.7)	171	31	5.5(4.7-6.3)
**Suicide, self-harm & injuries of undetermined Intent**	805	53	15.2(14.1-16.2)	485	39	12.4(11.3-13.5)	320	14	22.9(20.3-25.4)
**Other external cause**	28	6	4.7(2.9-6.4)	16	3	5.3 (2.7- 7.9)	12	3	4.0(1.7-6.3)
**Other natural diseases**	1,589	388	4.1(3.9-4.3)	686	159	4.3 (4.0-4.6)	903	229	3.9(3.7-4.2)

CI: Confidence interval; SMR: Standardised mortality ratio

**Conclusions:**

People hospitalised for depression continue to have higher all-cause mortality than the general population in Scotland, with relative mortality varying by cause of death.

**Disclosure of Interest:**

None Declared

